# Supplementary material for: Alcohol consumption drives sex- and region- specific disruption of somatostatin signaling in mice
Source: bioRxiv. 2025 Jun 17:2025.06.16.659907. Preprint. [Version 1] doi: 10.1101/2025.06.16.659907 (PMC12262583; doi:10.1101/2025.06.16.659907)
Supplement: 1 [file NIHPP2025.06.16.659907v1-supplement-1.pdf]

bioRxiv preprint doi: https://doi.org/10.1101/2025.06.16.659907; this version posted June 17, 2025. The copyright holder for this preprint (which was not certified by peer review) is the author/funder, who has granted bioRxiv a license to display the preprint in perpetuity. It is made available under aCC-BY 4.0 International license.

Alcohol consumption drives sex- and region- specific disruption of somatostatin signaling in mice

Running title: Alcohol and cortical somatostatin

Brockway et al.

Supplementary Methods

Exploratory behavior testing

24 hours post-DID, a short battery comprised of open field testing (OFT) and elevated plus maze (EPM) assessment was used to investigate changes in exploratory behavior after DID (**Supplementary Figures 1 and 2**). Tests were conducted in the dark phase of the daily light cycle under red light. Behavior was recorded by a camera mounted above the arenas. First, mice were transferred to a procedure room and allowed to acclimate for at least an hour before behavioral testing. Each mouse was placed in the corner of the open field (50 × 50 cm; black plexiglass 20 cm walls) and allowed to freely explore for 5 min. After testing, mice were returned to home cages and were not disturbed for at least one hr. Next, each mouse was placed in the center of an elevated plus maze (40 cm elevation; 30 × 5 cm arms; clear plexiglass 20 cm walls on closed arms) and monitored for 5 min. Behaviors were tracked using DeepLabCut (Brockway et al., 2023) and statistical analysis was conducted in MATLAB. Generalized linear mixed-effect models were used to test the associations between exploratory behaviors with DID treatment assignment and, among alcohol-exposed subjects, total alcohol and last binge alcohol consumed. Behavior was tested in multiple cohorts, which was included as a random effect in the analysis. For assessment of DID treatment effects, the model was: [outcome variable] ~ 1 + DID*Sex + (1|Cohort). To assess total alcohol and last binge alcohol effects within alcohol-exposed groups, the model was: [outcome variable] ~ 1 + Total_EtOH*Sex + Last_Binge_EtOH + (1|Cohort).

Somatostatin immunohistochemistry for cell density

bioRxiv preprint doi: https://doi.org/10.1101/2025.06.16.659907; this version posted June 17, 2025. The copyright holder for this preprint (which was not certified by peer review) is the author/funder, who has granted bioRxiv a license to display the preprint in perpetuity. It is made available under aCC-BY 4.0 International license.

Mice were deeply anesthetized with Avertin (250 mg/kg) and perfused transcardially with ice-cold phosphate buffered saline (PBS, pH 7.4) and 4% paraformaldehyde (PFA, pH 7.4). Brains were removed, post-fixed in PFA overnight washed 3x in PBS and stored in PBS at 4 C for less than 1 week. 40-μm free floating sections containing the PFC were sliced with a vibrating microtome (Leica, VS 1200) and stored in PBS until staining, conducted within one week.

Prior to immunostaining, slices were washed 3 times with PBS for 10 min each on a Fisherbrand™ Multi-Platform Shaker, then underwent antigen retrieval in 10 mM sodium citrate buffer (pH 6.0) at 80°C for 30 min. Slices were washed three times in PBS for 10 min each, and permeabilized in 0.5% Triton X-100 in PBS for 60 min. Nonspecific binding was blocked with 5% normal goat serum (NGS) in 0.1% Triton X-100 in PBS for 60 min. Slices were washed 3 times in PBS for 10 min each, then incubated in a primary antibody rat anti-somatostatin (1:500, Millipore, Burlington, MA, United States) in 2.5% NGS in 0.1% Triton X-100 in PBS for 48-h at 4°C. Slices were washed three 3 times with PBS for 10 min each, and incubated in a fluorophore-tagged secondary antibody goat anti-rat Cy3 (1:500, Millipore, Burlington, MA, United States) for 4-h at room temperature. Slices were washed again three times with PBS, with the last step including DAPI (1:10,000). Slices were mounted on glass slides, air-dried and coverslipped with Immunomount (Thermo Fisher Scientific, Waltham, MA, United States). Images were obtained at 20x with an Olympus BX63 upright microscope (Center Valley, PA, United States) under matched exposure settings. Eight images from the PFC were taken per mouse.

SST+ cell counts were performed using ImageJ (National Institutes of Health, Bethesda, MD, United States). The anterior cingulate cortex (ACC), prelimbic (PL) cortex and infralimbic (IL) cortex were delineated and SST neurons were quantified in each region separately under matched criteria for size, circularity, and intensity consistent with our previously published work (Dao et al., 2020; Suresh Nair et al., 2022). The threshold for delineation of cells was set at 8.10±.01. Each ROI's total SST cell count was divided by the ROI area to give a total SST density represented in cells/mm^2. 5-8 sections containing the ACC, PL, and IL were quantified and averaged to obtain one value per mouse. Imaging, cell counting, and analysis was done blinded to condition and sex. Original images were used for quantification and representative images were adjusted for brightness and contrast.

bioRxiv preprint doi: https://doi.org/10.1101/2025.06.16.659907; this version posted June 17, 2025. The copyright holder for this preprint (which was not certified by peer review) is the author/funder, who has granted bioRxiv a license to display the preprint in perpetuity. It is made available under aCC-BY 4.0 International license.

RNAscope

Fresh-frozen brains were sliced in 20 μm coronal sections between coordinates 1.3 mm to 1.9 mm relative to Bregma to capture the PL cortex. Slides were prepared to include anatomically matched sections from each experimental group on each slide. RNAscope was performed on three slides for each animal, as previously published (Wang et al., 2012) using the RNAscope Multiplex Fluorescent v2 Assay kit. Slides were kept in 100% ethanol overnight at -20°C following the fixation and dehydration steps. Slides were then stained for SSTR4 using the RNAscope Probe-Mm-SSTR4-C2 (ACDBio, #416641-C2), diluted 1:50 in RNAscope Probe Dilutant (ACDBio, # 300041). Slides were counterstained with DAPI (provided in the RNAscope kit). Opal 690 (Akoya Biosciences, #FP1497001KT) was used for the SSTR4 probe. One image was taken from each hemisphere of using a STELLARIS 5 white light laser confocal microscope (Leica) with a 20X objective and 2X zoom. The pinhole, gain, and exposure were consistent for each image on a single slide. Images were taken 1024 x 1024 resolution with 2X line averaging. All staining and imaging were done by an experimenter blinded to the conditions.

RNAscope image processing

RNAscope images were segmented and measured in ImageJ using a custom Python library (**Supplementary Figure 3**). The semi-automated pipeline required user inspection of each individual image, and selection of a few key parameters to be used in fully automated processing for the second stage. First, each two-channel color confocal image was separated into DAPI and SSTR grayscale channels, respectively. To avoid registration of single pixels, we used a small gaussian blur between 0 and 3 pixels in x and y directions on each image, determined manually by inspection. We found that DAPI image registration improved with gaussian blurs with 2-pixel radius, whereas SSTR images generally required less gaussian blur for accurate registration. These blurred images were then binarized using a threshold function with a range

bioRxiv preprint doi: https://doi.org/10.1101/2025.06.16.659907; this version posted June 17, 2025. The copyright holder for this preprint (which was not certified by peer review) is the author/funder, who has granted bioRxiv a license to display the preprint in perpetuity. It is made available under aCC-BY 4.0 International license.

manually selected to maximize retention of visible puncta, while minimizing the effect of spatially varying illuminance from uneven sample thickness or edge artifacts. The binarized DAPI images were processed with a “fill holes” function to close incompletely closed regions, and then a watershed function was used to segment adjoined DAPI regions. Each individual DAPI region was then labeled with an identifier unique to that image. We then saved the selected gaussian blur, watershed, and threshold parameters for each channel, in each image, and used a fully automated algorithm to complete segmentation and ROI measurement of all images using an ImageJ script. This algorithm recorded the area of each individual DAPI region and the area of the overlapping SSTR puncta, then saved these results in a tabulated comma-delimited file, along with accompanying file-specific metadata. All comma-delimited files were then opened, joined and analyzed in R using the ESS package in GNU-Emacs.

After unblinding to animal sex and alcohol condition, we asked whether our manually selected image parameters were randomly biased across these factors by chance. To test if manually-selected gaussian blur values or threshold values varied with animal sex, or alcohol condition, we performed 2-way ANOVA with main effects Sex and Group and random effects Animal ID on these dependent variables. We found that gaussian blur radii in DAPI images were no different between sexes or experimental group (Sex, $F[1,11.2]=1.0$, $p=0.34$; Group, $F[1,11.2]=0.04$ $p=0.82$). Likewise, gaussian blur parameters selected for SSTR images were the same between mouse sexes and groups (Sex, $F[1,10.9]=0.45$, $p=0.51$; Group, $F[1,10.9]=0.09$, $p=0.75$). We also tested whether manually-selected threshold values were different between mouse sexes or treatment arms in both DAPI and SSTR images. In DAPI images, the manually selected threshold values did not differ between animal sex or treatment arm (Sex, $F[1,532]=0.12$, $p=0.72$; Group, $F[1,532]=0.11$, $p=0.74$). The same was true for threshold values in SSTR images (Sex, $F[1,11.9]=1.01$, $p=0.33$; Group, $F[1,11.9]=0.24$, $p=0.62$; **Supplementary Figure 4**). Thus, manually selected image processing parameters were not different between images from male or female mice, nor between images of ETOH+ and control mice.

We then quantified DAPI registration errors across images. Registration error occurs when regions of extreme size are incorrectly identified as a DAPI region, caused at the extremes by single-pixel registrations or registration of large, improperly segmented regions. To identify registration errors, we log-transformed the experiment-wise distribution of DAPI region area to approximate normality, then defined outliers as DAPI

bioRxiv preprint doi: https://doi.org/10.1101/2025.06.16.659907; this version posted June 17, 2025. The copyright holder for this preprint (which was not certified by peer review) is the author/funder, who has granted bioRxiv a license to display the preprint in perpetuity. It is made available under aCC-BY 4.0 International license.

regions with area more extreme than 1.5 IQR of the median (**Supplementary Figure 4C-D**). Using this inclusion criterion, we found no difference in the outlier rate based on animal sex (F[1,11.0]=2.29, p=0.15) or treatment arm (F[1,11.0]=1.88, p=0.19). Nearly all excluded regions were below the minimum acceptable DAPI size (<3.68 m2), consistent with the left-skewedness of the log-transformed distribution. This distribution is expected after segmenting DAPI images with a watershed function, as the watershed disproportionately splits larger DAPI regions into smaller ones, skewing the distribution left.

Patch-clamp slice electrophysiology

Brains were immediately placed in ice-cold oxygenated NMDG-HEPES artificial cerebrospinal fluid (aCSF) containing the following, in mM: 92 NMDG, 2.5 KCl, 1.25 NaH_2PO_4, 30 $NaHCO_3$, 20 HEPES, 25 glucose, 2 thiourea, 5 Na-ascorbate, 3 Na-pyruvate, 0.5 $CaCl_2 \cdot 2H_2O$, and 10 $MgSO_4 \cdot 7H_2O$ (pH to 7.3–7.4). The PL was identified according to the Allen Mouse Brain Atlas. 300 µm coronal slices containing the PL were prepared on a Compresstome Vibrating Microtome VF-300-0Z (Precisionary Instruments, Greenville, NC), and transferred to heated (31°C) NMDG-HEPES (in mM: 124 NaCl, 4.4 KCl, 2 $CaCl_2$, 1.2 $MgSO_4$, 1 NaH_2PO_4, 10.0 glucose, and 26.0 $NaHCO_3$, pH 7.4, mOsm 300-310), for a maximum of 10 min. Slices were then transferred to heated (31°C) oxygenated normal aCSF where they were allowed to rest for at least 1 hr before use. Finally, slices were moved to a submerged recording chamber where they were continuously perfused with the recording aCSF (2 mL per min flow rate, 31°C). Recording electrodes (3–6 MΩ) were pulled from thin-walled borosilicate glass capillaries with a Narishige PC-100 Puller.

Following rupture of the cell membrane, cells were held in current-clamp. A minimum of 5 min stable baseline was acquired prior to experiments and bath application. Measurements of intrinsic excitability were conducted at both resting membrane potential (RMP) and at the standard holding potential of –70 mV both before and after application. Gap-free RMP was recorded during the entire drug application period. For optogenetic activation of SST cells with simultaneous electrophysiological recording of pyramidal cells in layer 2/3 of the PL cortex, slices from SST-IRES-Cre:Ai32 mice (expressing Cre-dependent channelrhodopsin in SST cells) were kept shielded from light, and experiments performed under low illumination. 25 µM Picrotoxin

bioRxiv preprint doi: https://doi.org/10.1101/2025.06.16.659907; this version posted June 17, 2025. The copyright holder for this preprint (which was not certified by peer review) is the author/funder, who has granted bioRxiv a license to display the preprint in perpetuity. It is made available under aCC-BY 4.0 International license.

(Hello Bio, HB0506), and 1 μM CGP 55845 (Tocris, 1248) were added to the aCSF to block GABAergic signaling. Cells were held in current clamp and membrane potential (mV) was measured. Following establishment of a 5 min stable baseline, a 470-nm LED (CoolLED, United Kingdom) was directed to the slice for 10 min (10 Hz frequency).

Enzyme immunoassay for photostimulated release of SST

24 hr after DID, SST-IRES-Cre:Ai32 mice (expressing Cre-dependent channelrhodopsin in SST cells) were sacrificed for enzyme immunoassay for photostimulated release of SST. Briefly, mice were rapidly decapitated under isofluorance anesthesia and the dissected brain was placed in ice-cold modified high-sucrose artificial cerebrospinal fluid (in mM: 194 sucrose, 20 NaCl, 4.4 KCl, 2 CaCl2, 1 MgCl2, 1.2 NaH2PO4, 10.0 glucose, and 26.0 NaHCO3, pH 7.4). 150 mM coronal slices containing the PL were sectioned on a Compresstome (Precisionary Instruments, Greenville, NC) and transferred to normal aCSF (in mM: 124 NaCl, 4.4 KCl, 2 CaCl2, 1.2 MgSO4, 1 NaH2PO4, 10.0 glucose, and 26.0 NaHCO3, pH 7.4) for 30 min. Slices were maintained in a holding chamber at 31 °C, continuously bubbled with a 95% O2/ 5% CO2 mixture and shielded from light until photostimulation. Individual slices were placed in a well on a 12-well plate with 500 mL of oxygenated normal aCSF. New aCSF was used at this step to control for any SST leakage following slicing. A 470-nm laser (CoolLED, United Kingdom) was directed to the PL cortex for 15 min at a stimulation frequency of 10 Hz. Stimulation/control conditions were counterbalanced between rostral and caudal PL slices to account for variability in viral expression along the anterior/posterior axis. No peptidase enzyme inhibitor was added to the surrounding aCSF. Samples were kept on ice until processed for enzyme linked immunoassay (ELISA).

At the end of the experiment, 2 x 50 mL of the aCSF within the well was pipetted into a 96-well SST ELISA plate (LSBio Somatostatin ELISA Kit LS-F12622) and ELISA (including standard curve) were performed identical to manufactures instructions. The assay is specific for mature forms of SST, somatostatin-14 and somatostatin-28, with no cross-reaction to similar peptides such as neuropeptide Y and vasoactive intestinal peptide. Absorbance was read at 450 nm on a Synergy2 microplate reader (BioTek, Winooski, VT). SST release was quantified in pg/mL. Following optical stimulation, slices were visualized under infrared video

bioRxiv preprint doi: https://doi.org/10.1101/2025.06.16.659907; this version posted June 17, 2025. The copyright holder for this preprint (which was not certified by peer review) is the author/funder, who has granted bioRxiv a license to display the preprint in perpetuity. It is made available under aCC-BY 4.0 International license.

microscope and blue LED (470 nm) for verification of viability and eYFP expression in the PL. Photostimulation, ELISA, and analysis were performed blinded to condition.

bioRxiv preprint doi: https://doi.org/10.1101/2025.06.16.659907; this version posted June 17, 2025. The copyright holder for this preprint (which was not certified by peer review) is the author/funder, who has granted bioRxiv a license to display the preprint in perpetuity. It is made available under aCC-BY 4.0 International license.

Supplementary Figures

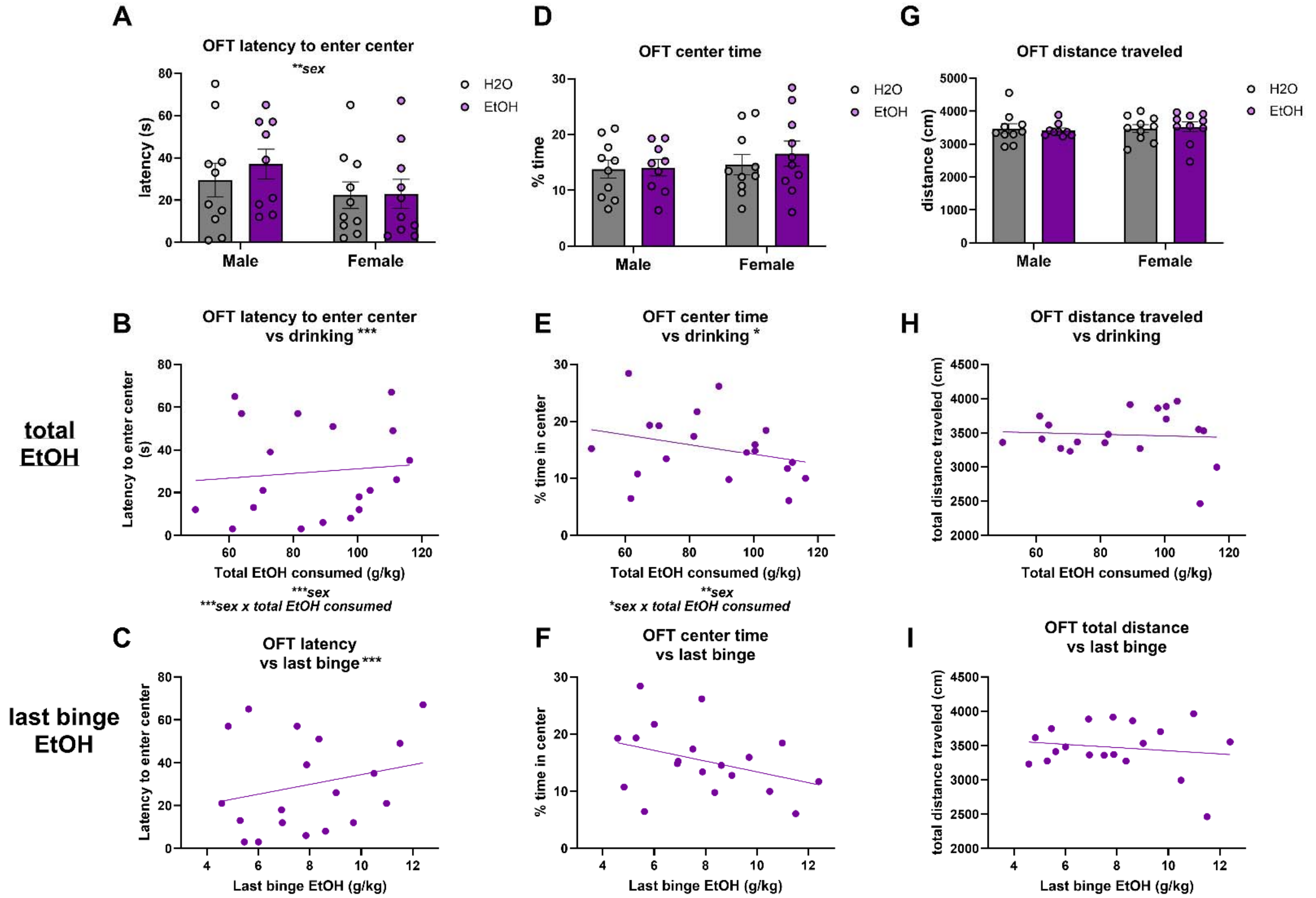

Supplementary Figure 1. Exploratory behaviors in the OFT 24 hours post-DID. Among DID mice, total and last binge alcohol consumption were inversely associated with exploratory behaviors 24 hr post-DID. (A) Across all conditions, latency to enter the center was higher in males than females (male: 0.2798 ± 0.088736, p = 0.003). (B-C) Total alcohol consumed (0.05166 ± 0.0095662, p<0.001) and last binge alcohol consumed (0.16692 ± 0.036066, p<0.001) were positively associated with latency to enter center. Again, males exhibited a longer latency to enter the center than females (male: 7.0737 ± 0.99888, p<0.001). An interaction of sex and total alcohol consumed was also detected (male x total EtOH: -0.059403 ± 0.0097241, p<0.001). (D) No effects of DID or sex were detected. (E-F) Total alcohol consumed was inversely associated with time spent in the center (-0.30748 ± 0.1125, p=0.016), which was also influenced by an interaction of sex and total alcohol consumed (male x total EtOH: 0.3369 ± 0.12171, p=0.015). Independent of alcohol effects, males spent less time in the center than females (male: -36.529 ± 10.263, p = 0.003). (G-I) Total distance traveled in the open field was not found to be influenced by sex or alcohol. For scatterplots, lines of best fit were added independently of the described analysis to aid visualization. * = p<0.05, ** = p<0.01, *** = p<0.001

bioRxiv preprint doi: https://doi.org/10.1101/2025.06.16.659907; this version posted June 17, 2025. The copyright holder for this preprint (which was not certified by peer review) is the author/funder, who has granted bioRxiv a license to display the preprint in perpetuity. It is made available under aCC-BY 4.0 International license.

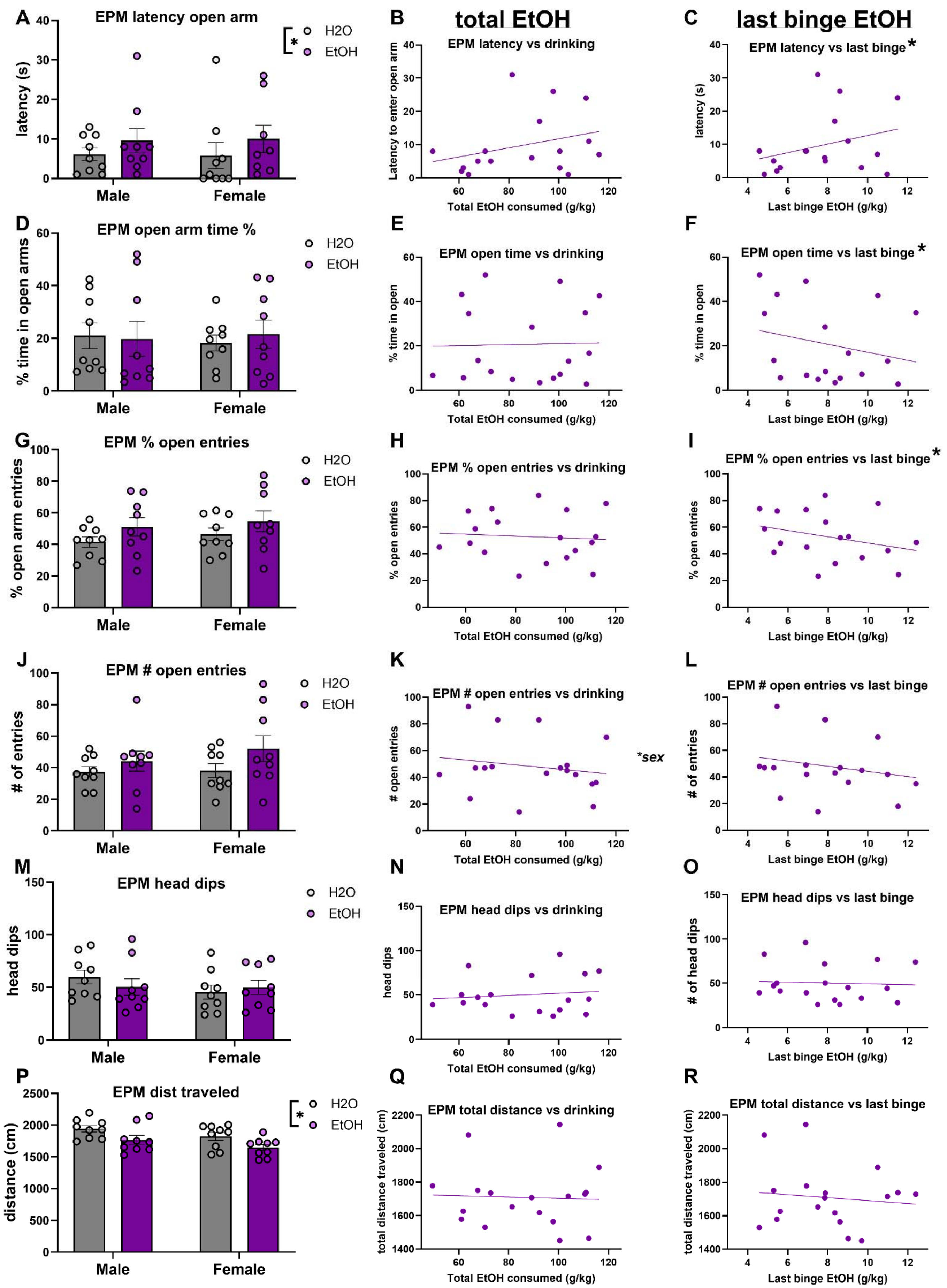

bioRxiv preprint doi: https://doi.org/10.1101/2025.06.16.659907; this version posted June 17, 2025. The copyright holder for this preprint (which was not certified by peer review) is the author/funder, who has granted bioRxiv a license to display the preprint in perpetuity. It is made available under aCC-BY 4.0 International license.

Supplementary Figure 2. Exploratory behaviors in the EPM 24 hours post-DID. Alcohol exposure reduced exploratory behaviors in the elevated plus maze 24 hr post-DID, and last binge alcohol consumption strongly predicted decreases in exploration. (A-C) Mice that consumed alcohol had an increased latency to enter an open arm relative to water-exposed control subjects (EtOH: 0.50305 ± 0.17824, p=0.008). Among alcohol-exposed subjects, alcohol consumed during the last binge was positively associated with latency to enter an open arm (0.19367 ± 0.078624, p=0.030). (D-F) There were no differences in percent time spent in open arms between alcohol- and water-exposed controls, but within alcohol-exposed subjects, alcohol consumed during the last binge was negatively associated with time in open arms (-6.9308 ± 3.1176, p=0.045). (G-I) Similarly, percent open arm entries were similar between alcohol- and water-exposed groups, but last binge alcohol was negatively associated with percent open arm entries (-7.5471 ± 2.6988, p=0.015). (J-L) No influence of alcohol exposure was detected on the number of open arm entries, but females entered open arms more frequently than males (male: -114.73 ± 46.288, p=0.028). (M-O) Number of head dips was not dependent upon alcohol exposure variables or sex. (P-R) Subjects that had access to alcohol traveled a shorter distance than water-exposed control subjects (EtOH: -174.94 ± 77.49, p=0.031), although this did not vary by total or last binge alcohol consumption among alcohol-exposed subjects. For scatterplots, lines of best fit were added independent of the described analysis to aid visualization. * = p<0.05, ** = p<0.01, *** = p<0.001

bioRxiv preprint doi: https://doi.org/10.1101/2025.06.16.659907; this version posted June 17, 2025. The copyright holder for this preprint (which was not certified by peer review) is the author/funder, who has granted bioRxiv a license to display the preprint in perpetuity. It is made available under aCC-BY 4.0 International license.

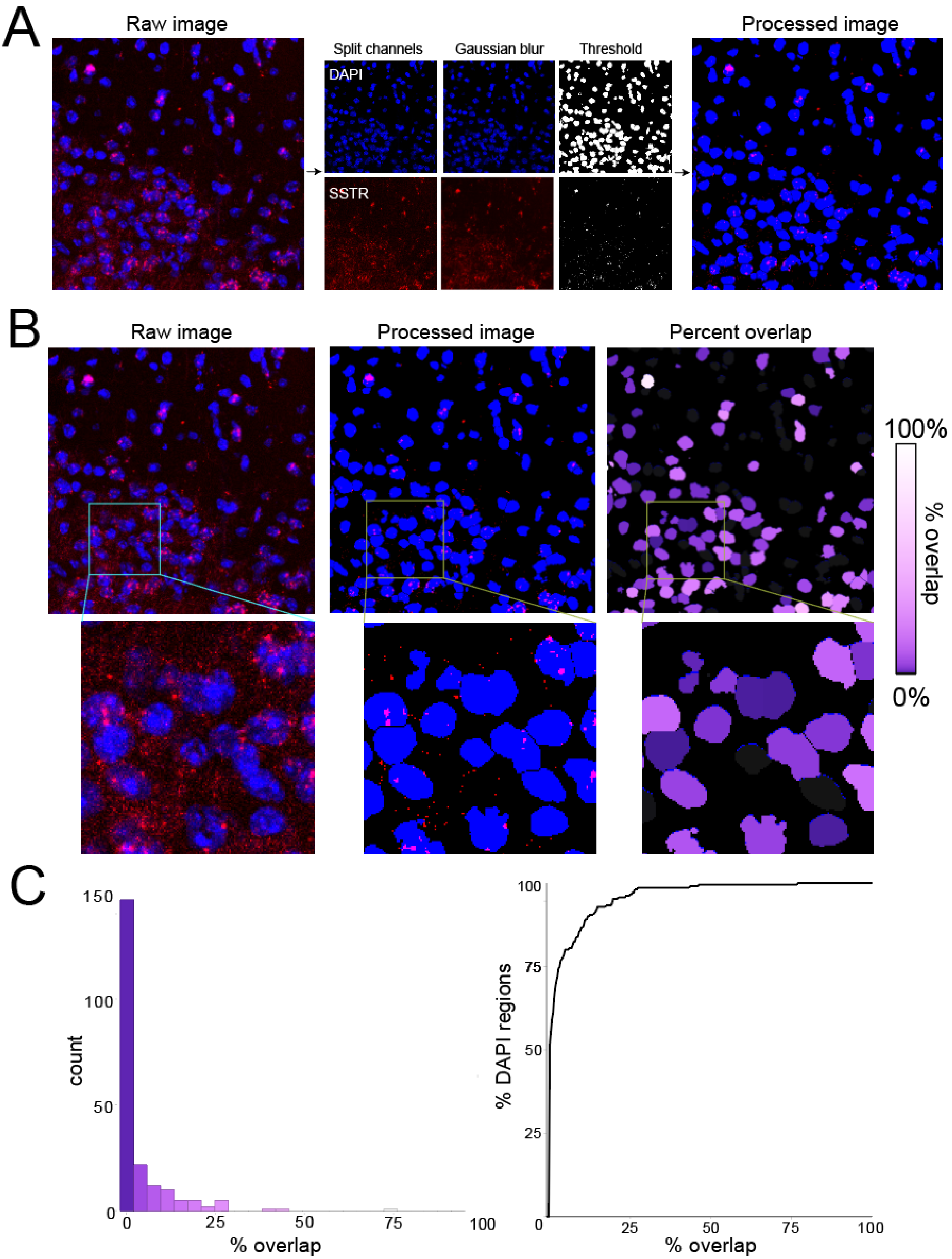

L3

bioRxiv preprint doi: https://doi.org/10.1101/2025.06.16.659907; this version posted June 17, 2025. The copyright holder for this preprint (which was not certified by peer review) is the author/funder, who has granted bioRxiv a license to display the preprint in perpetuity. It is made available under aCC-BY 4.0 International license.

Supplementary Figure 3. Image processing pipeline. (A) Schematic of the image processing algorithm used to identify DAPI regions and SSTR puncta. (B) Close-ups of raw, processed, and overlap-measured images, with accompanying insets. (C) Histogram and cumulative distribution function of overlap-measured image in B.

bioRxiv preprint doi: https://doi.org/10.1101/2025.06.16.659907; this version posted June 17, 2025. The copyright holder for this preprint (which was not certified by peer review) is the author/funder, who has granted bioRxiv a license to display the preprint in perpetuity. It is made available under aCC-BY 4.0 International license.

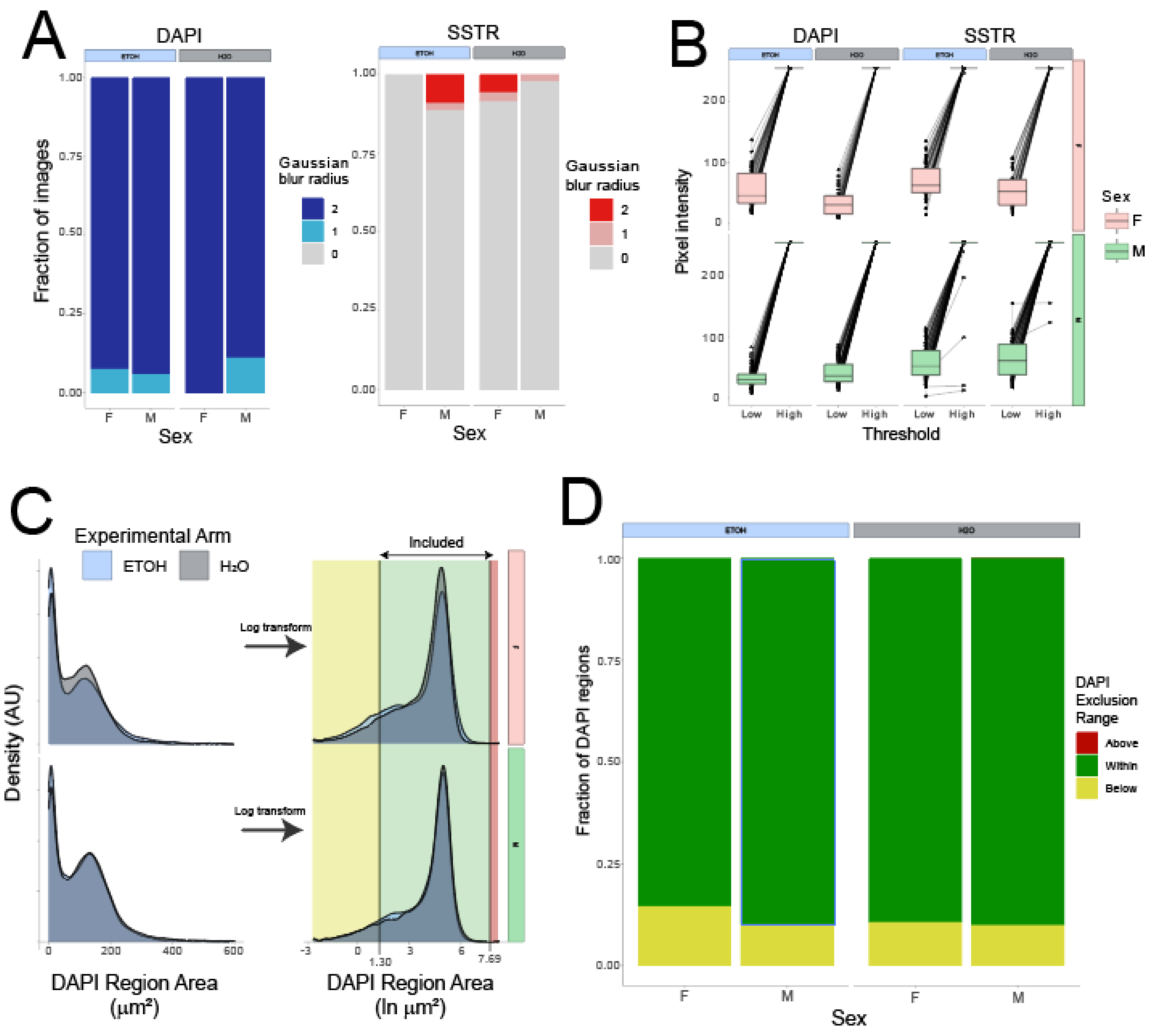

bioRxiv preprint doi: https://doi.org/10.1101/2025.06.16.659907; this version posted June 17, 2025. The copyright holder for this preprint (which was not certified by peer review) is the author/funder, who has granted bioRxiv a license to display the preprint in perpetuity. It is made available under aCC-BY 4.0 International license.

Supplementary Figure 4. Descriptive statistics from experiment-wise image registration. (A) Manual selection of gaussian blur radius, in both DAPI and SSTR channel0, shown across experimental factors. (B) Manual selection of low and high pixel threshold values for DAPI and SSTR channels, shown across experimental factors. (C) Experiment-wise distribution of DAPI region areas, before and after log-transform, with inclusion range used across the experiment. (D). DAPI region exclusion shown across experimental factors.
